# DNA methyltransferases and TETs in the regulation of differentiation and invasiveness of extra-villous trophoblasts

**DOI:** 10.3389/fgene.2013.00265

**Published:** 2013-12-04

**Authors:** Philip C. Logan, Murray D. Mitchell, Peter E. Lobie

**Affiliations:** ^1^The Liggins Institute, The University of AucklandAuckland, New Zealand; ^2^University of Queensland Centre for Clinical Research, University of QueenslandBrisbane, QLD, Australia; ^3^Cancer Science Institute of Singapore, National University of SingaporeSingapore, Singapore

**Keywords:** DNA methyltransferases, trophoblasts, cell differentiation, TETs, placenta, chromatin condensation, hydroxymethylation, epigenetics

## Abstract

Specialized cell types of trophoblast cells form the placenta in which each cell type has particular properties of proliferation and invasion. The placenta sustains the growth of the fetus throughout pregnancy and any aberrant trophoblast differentiation or invasion potentially affects the future health of the child and adult. Recently, the field of epigenetics has been applied to understand differentiation of trophoblast lineages and embryonic stem cells (ESC), from fertilization of the oocyte onward. Each trophoblast cell-type has a distinctive epigenetic profile and we will concentrate on the epigenetic mechanism of DNA methyltransferases and TETs that regulate DNA methylation. Environmental factors affecting the mother potentially regulate the DNA methyltransferases in trophoblasts, and so do steroid hormones, cell cycle regulators, such as p53, and cytokines, especially interlukin-1β. There are interesting questions of why trophoblast genomes are globally hypomethylated yet specific genes can be suppressed by hypermethylation (in general, tumor suppressor genes, such as E-cadherin) and how invasive cell-types are liable to have condensed chromatin, as in metastatic cancer cells. Future work will attempt to understand the interactive nature of all epigenetic mechanisms together and their effect on the complex biological system of trophoblast differentiation and invasion in normal as well as pathological conditions.

## Epigenetic mechanisms are involved in developing a healthy placenta

**Figure 1:**
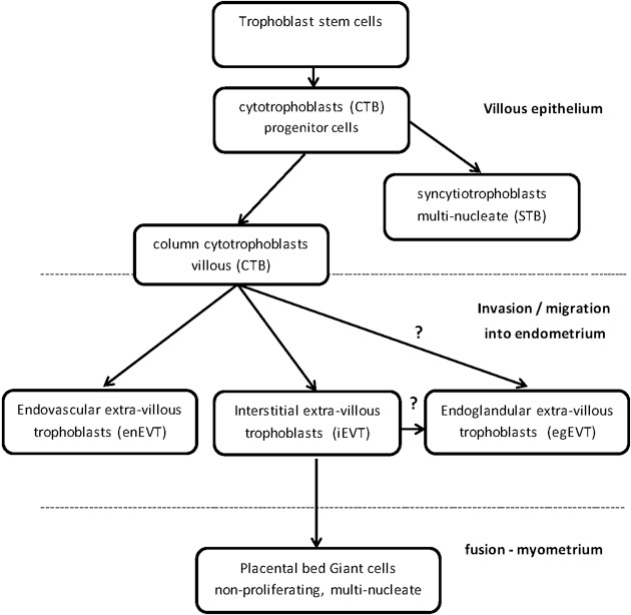
**Trophoblast stem cells differentiate into specialized trophoblast cells**.

The epigenetic mechanism of DNA methylation partially regulates trophoblast differentiation and invasion into the endometrium of the uterus in order to establish and maintain a healthy placenta for the growing fetus. The placenta is an essential organ for sustaining the life of the fetus through which nutrients, oxygen exchange, immune barrier protection, and waste disposal are achieved between the fetal and maternal blood circulations (Cross et al., 1994; Arck and Hecher, 2013). Defective placentation and aberrant differentiation and invasion of the trophoblasts potentially results in pathologies of pregnancy, which include pre-eclampsia, intrauterine growth restriction (IUGR) (Maccani and Carmen, 2009; Kokkinos et al., 2010), spontaneous abortion, preterm birth (Khong and Brosens, 2011), placenta acreta, “Hemolysis, Elevated Liver enzymes, Low Platelets” (HELLP) (van Dijk et al., 2012), and choriocarcinoma (Graham and Lala, 1992; Norwitz, 2006). Environmental factors, such as diet and oxygen stress, can adversely affect the epigenetic mechanisms that the embryo relies on to implant and fully grow into a healthy fetus (Yuen et al., 2013). Epigenetic mechanisms partially regulate extra-villous trophoblast differentiation and invasiveness into the endometrium (Figure 1) (Rahnama et al., 2006; Rugg-Gunn, 2012; Chen et al., 2013a). Over the last decade the study of epigenetics has been applied to many complex biological systems, such as trophoblast differentiation, in order to reach an understanding of the mechanisms and the pathways (Cox et al., 2009, 2011; Choi, 2010; Hemberger, 2010; Senner and Hemberger, 2010; Turner et al., 2012; Arnold et al., 2013).

## DNA methylation is one of several epigenetic mechanisms

DNA methylation, histone modification and non-coding RNA (ncRNA) and the interactions between them are some of the epigenetic mechanisms that have been studied in the placenta (Fuks et al., 2003; Nelissen et al., 2011). The field of epigenetics is continually expanding and there is recent interest in DNA hydroxymethylation and TET methylcytosine dioxygenases (ten-eleven translocation) (Tahiliani et al., 2009; Dahl et al., 2011; Xu et al., 2011; Zhao and Chen, 2013), and the role of various types of ncRNA, such as micro RNAs (miRNA) (Tsai et al., 2009; Ji et al., 2013; Mouillet et al., 2013) and long intergenic ncRNA (lincRNA) (van Dijk et al., 2012). The three main DNMTs that methylate cytosines in CpG dinucleotides to 5-methylcytosine (5mC) are DNMT1, the maintenance methyltransferase, and the *de novo* methyltransferases DNMT3A and DNMT3B, which each have gene-specific methylation sites in distinct genomic DNA regions (Hsieh, 1999; Okano et al., 1999; Takeshima et al., 2006). Another member of the DNMT3 family is DNMT3L, which is essential for placental development through establishing maternal gene imprinting (Chédin et al., 2002; Hata et al., 2002; Arima et al., 2006). Imprinting of paternal or maternal genes has an important effect on placenta gene expression and subsequent growth of the fetus (Reik and Walter, 2001; Wang et al., 2013a). DNA methylation of CpGs generally silences a gene by at least three different functions; the binding of transcription factors (Watt and Molloy, 1988), attracting methyl binding domain proteins (MBD) (Boyes and Bird, 1991), and altering chromatin packaging (Ng and Bird, 1999). Moreover, DNMTs have both DNA methylation-independent as well as methylation-dependent functions (Bachman et al., 2001; Fuks et al., 2001; Esteve et al., 2005; Pavlopoulou and Kossida, 2010). There are a few instances when instead of repressing transcription, DNA methylation activates transcription, but how this is achieved is still unknown, but release of inhibitors is most likely (Gellersen and Kempf, 1990; Niesen et al., 2005). TETs are involved in an active DNA demethylation pathway, by converting 5mC to 5-hydroxymethylcytosine (5hmC) (Seisenberger et al., 2013). These epigenetic mechanisms critically regulate the differentiation of cells essential for the development of the placenta and embryo (Table 1) (Ohgane et al., 2002; Rugg-Gunn, 2012).

**Table 1:** **DNA methyltransferases (DNMTs) and TETs regulate specific key genes for differentiation and invasion**.

**Cell type**	**DNMTs and TETs**	**Specific genes regulated by DNA methylation**
Trophoblast stem cells Cytotrophoblasts (CTB)	↓DNMT1, TET1, 2	↑*hCG*, *DNMT3L, C19MC miRNA*, *ELF5*
	↑DNMT3L, 3A, 3B (Arima et al., 2006; Novakovic et al., 2010; Ficz et al., 2011; Haouzi et al., 2011; Koh et al., 2011)	↓*POU5F1*, *DNMT1, Nanog*, *CYP24A1* (Hattori et al., 2004; Ng et al., 2008; Novakovic et al., 2009; Tsai et al., 2009; Novakovic et al., 2010; Senner, 2011; Grigoriu et al., 2011; Oda et al., 2013)
Syncytiotrophoblasts (STB)	↓DNMT1, 3A, 3B (Oda et al., 2013)	↑syncytin-1 (Hemberger, 2010)
Extravillous trophoblasts (iEVT, enEVT, egEVT?)	DNMT1	↑*Snail*, *Slug*, *REN*, *AGT*, *IL-1β*? (Pringle et al., 2011; Chen et al., 2005; Hashimoto et al., 2013)
	↑DNMT3A, 3B (Rahnama et al., 2006; Novakovic et al., 2010)	↓E-cadherin, *JUP*, *KRT7*, *RASSF1A*, *APC*, *SFRP2*, *WIF1*, *EN1*, maspin? (Sato et al., 2003; Rahnama et al., 2006; Novakovic et al., 2008; Shi et al., 2012)
Epithelial endometrial	↓DNMT1, 3A, 3B (Rahnama et al., 2009)	↑E-cadherin, *JUP* (Rahnama et al., 2009)
Endometrial cancer	↓TET1	↓*HAND2*, E-cadherin, *MLH1*, *HOXA11*, miRNAs (Simpkins et al., 1999; Baylin et al., 2001; Saito et al., 2003; Whitcomb et al., 2003; Jones et al., 2013; Li et al., 2013)
	↑DNMT1, 3B (Beaulieu et al., 2002; Jin et al., 2005; Liao et al., 2008; Cui et al., 2009; Hsu et al., 2012; Kudo et al., 2012)	
Endometriotic	↑DNMT1, 3A, 3B (Wu et al., 2007)	↓*HOXA10*, *PRB, miR-196b* (Guo, 2009; Abe et al., 2013)
		↑*ERβ*, *NR5A1*
Embryonic stem (ESC)	-DNMT1, 3A, 3B, TET1, 2 (Li et al., 1992; Okano et al., 1999; Ficz et al., 2011; Ruzov et al., 2011; Dawlaty et al., 2013; Oda et al., 2013; Pastor et al., 2013)	↑syncytin-1, *ELF5* (Hattori et al., 2004; Matousková et al., 2006; Ng et al., 2008; Hemberger, 2010)-*POU5F1*

AGT, angiotensinogen; APC, adenomatous polyposis coli; C19MC miRNA, chromosome 19 miRNA cluster; CTB, cytotrophoblast; *CYP24A1*, vitamin D 24-hydroxylase; *DNMT*, DNA methyltransferase; egEVT, endoglandular extravillous trophoblast; *ELF5*, E74-like factor 5 (ets domain transcription factor); *EN1*, engrailed homeobox 1; enEVT, endothelial extravillous trophoblast; *ERβ*, estrogen receptor β; ESC, embryonic stem cell; HAND2, heart & neural crest derivatives expressed 2; *hCG*, human chorionic gonadotropin; *HOXA10/11*, homeobox A10/11; *IL-1β*, interlukin 1β; iEVT, interstitial trophoblast; *JUP*, plakoglobin, gamma catenin; *KRT7*, keratin 7; *MLH1*, mutL homolog 1; *NR5A1*, nuclear receptor subfamily 5, group A1 (steroidogenic factor 1); *POU5F1*, POU class 5 homeobox 1 (Oct4, octomer binding protein 4); *PRB*, progesterone receptor B; *RASSF1A*, Ras association domain family 1A; *REN*, prorenin; *SFRP2*, secreted frizzle-related protein 2; STB, syncytiotrophoblast; *TET*, ten-eleven translocation; *WIF1*, WNT inhibitory factor 1; ↓down-regulated; ↑over-expressed.

## DNA methylation maintains distinct stem cell lineages

DNA methylation is involved in the distinction between the specialized cell lineages of pregnancy beginning from fertilization until term (Hemberger, 2007; Nelissen et al., 2011; Xu et al., 2011). The blastocyst has embryonic stem cell (ESC) lineages in the inner cell mass, which will form the embryo, and trophoblast stem cells in the trophectoderm of extra-embryonic cells, which will develop into the placenta (Bischof and Irminger-Finger, 2005). Histone modifications are thought to establish the lineages of inner cell mass and trophectoderm (Nakanishi et al., 2012) and then DNA methylation profiles “lock in” and maintain the lineages to create a barrier between them, i.e., to block ESC from becoming trophoblast lineage (Ng et al., 2008; Senner, 2011; Oda et al., 2013). Both these lineages have the defining feature of a distinctive global DNA methylation pattern (Senner et al., 2012), so that the genome of the embryo is highly methylated, whilst the genome of the placental trophectoderm lineage of trophoblasts is globally hypomethylated (Hemberger, 2010; Xie et al., 2013). In the trophoblasts, DNMT1 is down-regulated by promoter methylation whereas DNMT3L is up-regulated, and in turn activates both DNMT3A and DNMT3B (Table 1) (Chédin et al., 2002; Hata et al., 2002; Suetake et al., 2004; Chen et al., 2005; Novakovic et al., 2010; Haouzi et al., 2011). A complex biological system, such as differentiating the trophoblast stem cell lineage, builds in redundancy. If DNMT1 is not expressed then DNMT3A and DNMT3B can substitute for DNMT1, if necessary, and maintain the methylation of DNA during cell proliferation (Liang et al., 2002; Walton et al., 2011; Arand et al., 2012). In a mouse triple knockout of DNMT1/3a/3b, extra-embryonic stem cells survived and proliferated whereas ESC either died or with minimal CpG methylation occasionally differentiated into trophoblast-like cells (Li et al., 1992; Jackson et al., 2004; Tsumura et al., 2006; Ng et al., 2008; Sakaue et al., 2010). However, knocking out DNMT3L disrupted the placenta in mice (Hata et al., 2002; Arima et al., 2006; Nelissen et al., 2011). Another feature of trophoblasts is the overall low level of 5hmC in the genome compared with the high level of 5hmC in the ESC genome (Ito et al., 2010; Ruzov et al., 2011).

## Pluripotency genes and TETs

To differentiate the trophoblast stem cell lineage from the ESC, the pluripotency, transcription factor gene, *POU5F1* (*OCT4*) is hypermethylated and repressed in trophoblast stem cells (Hattori et al., 2004; Li et al., 2007; Zhang et al., 2008; Zafarana et al., 2009; Senner, 2011). There are more hypermethylated promoters in trophoblast stem cells that silence pluripotency genes than in ESC whereas in ESC pluripotency-related genes are generally hypomethylated and expressed (Farthing et al., 2008). POU5F1 up-regulates TET1/2 in ESC to induce a high level of 5hmC and therefore pluripotency (Ruzov et al., 2011), on the other hand depletion of POU5F1 in trophoblast stem cells inhibits TET1/2 gene expression, which decreases 5hmC and pluripotency (Koh et al., 2011). TET1/2 depletion hypermethylates specific genes and if this occurs in ESC then pluripotency-related genes are down-regulated and this tends to differentiate ESC into extra-embryonic cells (Ficz et al., 2011; Koh et al., 2011; Williams et al., 2012). In humans, mutated, inactive TET2 can lead to cancers, such as hematopoietic cancer (Salker et al., 2011; Kudo et al., 2012; Perez et al., 2012), yet TET1/2 knockout mice can survive with reduced 5hmC. Perhaps the TET1/2 knockout mice survive because TET3 was increased and substituted for TET1/2, but the increased methylation compromised imprinted genes, and there was also an increase in extra-embryonic stem cells (Dawlaty et al., 2011, 2013). In a microarray study of TET1 knockout mice, 221 genes, mainly developmental, were down-regulated (Dawlaty et al., 2011). Depleted TET1 in stem cells up-regulate *ELF5*, the key trophoblast lineage-enforcing gene, which is hypomethylated and expressed in trophoblast stem cells but in ESC *ELF5* must be methylated and silenced (Ng et al., 2008; Senner and Hemberger, 2010; Hemberger, 2010; Senner, 2011). If ESC are induced to express ELF5 then ESC can become trophoblast-like (Roper and Hemberger, 2009; Koh et al., 2011).

## Trophoblast stem cells differentiate into specialised trophoblast cells

DNMTs and TETs are involved in the differentiation and regulation of the specialized trophoblast cells in the placenta (Table 1). Trophoblast stem cells are differentiated into proliferating, polarized epithelial cell cytotrophoblasts (CTB) and thence into villous CTB of anchoring villi and non-proliferating syncytiotrophoblasts (STB) (Figure 1) (Bischoff et al., 2000; Bischof and Irminger-Finger, 2005; Ji et al., 2013). The down-regulation of DNMT1, 3A and 3B in CTB hypomethylates the retroviral, fusogenic-protein, syncytin-1, and the consequent high expression of syncytin-1 differentiates the CTBs into multi-nucleated, single cell STB (Blond et al., 2000; Hemberger, 2010). Failure of this differentiation into STBs may result in pre-eclampsia or IUGR, with a different pattern of DNMTs and MBD proteins for each condition (Baczyk et al., 2009; Ruebner et al., 2013). For instance, the over-expression of DNMT3A will inhibit syncytin-1 gene expression and disrupt formation of STBs (Ruebner et al., 2013). The other pathway for CTB progenitor differentiation, from the anchoring villi columns, produces invasive intermediate trophoblasts (Fisher and Damsky, 1993), which invade the endometrium as either interstitial EVTs (iEVT) or endovascular-EVTs (enEVT) (Figure 1) (Pijnenborg et al., 1980). The intermediate EVTs also possibly invade uterine glandular cells as endoglandular EVTs (egEVT) in order to remodel and open up the uterine glands that can then secrete into the inter-villous space (Fitzgerald et al., 2010; Moser et al., 2010). The iEVTs continue to invade through the endometrium into the first third of the myometrium and in the placental bed become mononuclear cell aggregate and multi-nucleated giant cells (Fisher and Damsky, 1993; Al-Lamki et al., 1999). The enEVTs invade into the maternal spiral arteries that have been prepared for EVT invasion by decidual cells and uterine natural killer cells (Lash et al., 2010; Hannon et al., 2012) and by replacing the vascular endothelial and smooth muscle cells (Zhou et al., 1997), remodel these arteries into low pressure, high volume vessels capable of delivering sufficient maternal blood to the fetus through the placenta (Lockwood et al., 1999). The morphology and function of iEVTs and enEVTs are quite different to the STBs (Zhou et al., 1997).

## Epithelial cells transform into a highly invasive mesenchymal phenotype

To successfully become invasive EVTs, the CTBs undergo a transition from epithelial to highly invasive mesenchymal phenotype, called EMT (epithelial-mesenchymal transition), similar to invasive, metastatic cancer cells, except that EVTs are under strict spatial and temporal regulation (Bischof and Irminger-Finger, 2005; Perry et al., 2010; Apps et al., 2011). The decidual cells and extracellular matrix (ECM) inhibit the EVTs from invading too deeply (Bischoff et al., 2000). Recently, a microarray of first trimester trophoblasts identified over 3000 differentially regulated genes in the transition from villous CTB to EVT and this included *ELF5* that was down-regulated in EVT (Apps et al., 2011). In the EMT transition of epithelial CTBs into EVTs, the epithelial marker genes of E-cadherin and keratin 7 (*KRT7*) are hypermethylated and down-regulated (Rahnama et al., 2006; Chen et al., 2013a). Similar to invasive cancer cells, the tumor suppressor genes in EVTs are generally methylated, which down-regulates the genes, such as *APC*, E-cadherin, and possibly maspin (Rahnama et al., 2006; Wong et al., 2008; Shi et al., 2012). Two other EMT differentiation proteins, Snail and Slug, that are also involved in cancer and down-regulate E-cadherin, are up-regulated by hypomethylation in trophoblast cell-lines and differentiate CTBs into EVTs (Table 1) (Chen et al., 2013b). In endometrial cancer, an invasive phenotype is produced by an aberrant over-expression of DNMT1 and DNMT3B that down-regulates E-cadherin (Chan et al., 2003; Saito et al., 2003; Jin et al., 2005; Liao et al., 2008). In a similar epithelial-based invasive pathology, of endometriosis, DNMT1, 3A, 3B are over-expressed in the ectopic endometrial cells (Wu et al., 2007). DNMT1, 3A, 3B hypermethylate and down-regulate *PRB*, the progesterone receptor B, but there is no change in E-cadherin levels and so there is no EMT differentiation (Shaco-Levy et al., 2008). Concomitantly in endometriosis, DNMT1, 3A, 3B hypermethylate and down-regulate the anti-invasive *HOXA10* gene expression in the eutopic endometrium (Chu et al., 2004; Wu et al., 2005; Santamaria et al., 2012).

## Chromatin condensation correlates with invasiveness

Perhaps EVTs have chromatin condensation to enable migration and invasion? Although cancer cells generally have partially condensed chromatin between that of normal proliferative and senescent cells (Oh et al., 2013) there is a correlation between increased DNA hypermethylation, and therefore chromatin condensation, and increased invasiveness, which is essential during metastasis for the cancer cell intravasion into and extravasion out of blood vessels (Dufer et al., 2000; Fu et al., 2012). During cell migration the condensed chromatin allows the nuclei to be squeezed to fit through narrow gaps between cells (Gerlitz and Bustin, 2010; Fu et al., 2012). Constitutive heterochromatin is invariably condensed but when necessary the facultative heterochromatin can be condensed for invasion (Trojer and Reinberg, 2007). Chromatin condensation has also been observed in sperm, leukocytes, glioma cells, and neurons (Hammadeh et al., 1998; Gerlitz and Bustin, 2010). If cells are differentiated into invasive phenotypes then there is a tendency for the epigenetic mechanisms of DNA methylation and histone modification to condense the chromatin (Gerlitz and Bustin, 2010). When treatments of the histone methyltransferase inhibitor (5′-deoxy-5′-methyl-thioadenosine), HDAC inhibitors, or TETs were applied to invasive cell types then those cells decondensed the chromatin and invasiveness was inhibited (Gerlitz and Bustin, 2010; Fu et al., 2012; Song et al., 2013). In other cell types over-expression of TET increases 5hmC, which decreases 5mC (hypomethylation) and decondenses chromatin, so possibly the lack of TET expression in EVTs encourages an invasive phenotype by condensing the chromatin (Wanunu et al., 2010; Hsu et al., 2012; Pfeifer et al., 2013).

## DNA methylation regulates specific genes

Trophoblast genomes are globally hypomethylated, predominantly in repetitive DNA elements and intergenic regions, relative to somatic tissue genomes, yet concomitantly specific genes in trophoblasts are hypermethylated (generally tumor suppressor genes) to ensure EMT into EVTs and induce invasiveness similar to metastatic cancer cells (Gama-Sosa et al., 1983; Ehrlich, 2009; Apps et al., 2011; Novakovic and Saffery, 2012; Shen et al., 2012). Specific genes that are down-regulated by DNA methylation in EVTs have been identified and include E-cadherin, plakoglobin (*JUP*), *KRT7*, *DNMT1*, and five Wnt signaling inhibitors *APC*, *SFRP2*, *RASSF1A*, *EN1*, and *WIF1* (Table 1) (Rahnama et al., 2006; Chiu et al., 2007; Novakovic et al., 2008, 2010; Guilleret et al., 2009; Chen et al., 2013a). The increased Wnt signaling stimulates β-catenin accumulation in the nucleus, and that promotes EVT invasiveness (Pollheimer et al., 2006). Sometimes there is hypomethylation and up-regulation of specific genes in EVTs, such as the renin-angiotensin system genes (RAS) (Wang et al., 2012). In the immortalized EVT-like cell-line, HTR8/SVneo, treatments of both cyclic AMP and 5-aza-2′-deoxycytidine (AZA), an inhibitor of DNMTs and DNA methylation, up-regulated prorenin (*REN*), and angiotensinogen (*AGT*) (Wang et al., 2013b). RAS genes regulate EVT invasion and vascular remodeling and it has been suggested that aberrant expression could be linked to pre-eclampsia and IUGR (Williams et al., 2010; Pringle et al., 2011).

## Hypermethylation of specific genes concomitant with global hypomethylation

The question that has long been asked has been how do specific genes become hypermethylated and silenced in cells that otherwise have globally hypomethylated genomes, such as in cancer and now here in trophoblasts (Caiafa and Zampieri, 2005)? Global hypomethylation generally consists of vast hypomethylated regions of the genome that are depleted of genes whereas hypermethylated genes, such as tumor suppressor genes, have methylated promoters (Shen et al., 2012; Varley et al., 2013). A mouse tumor model showed that a functional DNMT3a determined that genomic hypomethylation was restricted to regional rather than the uniformly widespread hypomethylation throughout the genome that occurs in DNMT3a deficient mice (Raddatz et al., 2012). (This difference in DNMT3A expression could explain the possible difference between STB and EVT genomic hypomethylation.) DNA methylation should not be regarded as static in cancer because genomic hypomethylation and hypermethylation of specific genes increase progressively as the tumor increases in malignancy (Jones and Baylin, 2007; Esteller, 2008; Ehrlich, 2009) and even then not all tumor suppressor genes are hypermethylated (Hamilton et al., 2005). Regulation of hypermethylation of specific genes and genomic hypomethylation in cancer, and presumably in trophoblasts, is not dependent solely on DNMTs but involves the combined action with histone modification (Freitag and Selker, 2005; Esteller, 2008; Ehrlich, 2009), ncRNA (Kulis and Esteller, 2010), and TETs (Hsu et al., 2012; Williams et al., 2012). The inactivation of TETs can be involved in the targeted hypermethylation and silencing of specific tumor suppressor genes (Hsu et al., 2012; Williams et al., 2012). There is ample evidence that DNMTs interact with HDACs (Fuks et al., 2000; Bachman et al., 2001; Geiman et al., 2004) and transcription factors (Di Croce et al., 2002; Suzuki et al., 2005; Hervouet et al., 2009; Pavlopoulou and Kossida, 2010; Yuen et al., 2013), often in complexes, to target the methylation of specific genes (Robertson et al., 2000; Fuks et al., 2001; Zhang et al., 2005). Specific genes can be expressed in specific tissues (Schroeder et al., 2013), for example, syncytin-1 is highly expressed and tissue specific to the placenta (Mi et al., 2000; Matousková et al., 2006; Muir et al., 2006) however aberrant hypomethylation and expression of the syncytin-1 gene in the brain has been proposed to contribute to multiple sclerosis (Perron et al., 1997; Mattson and Taub, 2004).

## Factors that regulate the DNMTs

So what factors regulate the DNMTs? DNMTs can be regulated by steroid hormones (Cui et al., 2009; Yamagata et al., 2009; Vincent et al., 2011; Logan et al., 2013) growth factors (Shafiei et al., 2008), cell cycle regulators (Lin et al., 2010), viruses (Flanagan, 2006; Shamay et al., 2006), and cytokines (Karmakar and Das, 2002; Braconi et al., 2010). The cytokines of IL-1β, IL-6, VEGF, and cytokine-activated HIF-1α are involved in trophoblast invasion (Karmakar and Das, 2002; Dubinsky et al., 2010). IL-1β can regulate DNMTs, suppress methylation, reduce E-cadherin, stimulate MMP9 [crucial for EVT invasion (Whiteside et al., 2001)] and positively correlates with invasiveness (Librach et al., 1994; Karmakar and Das, 2004; Hashimoto et al., 2010; Nakano et al., 2013). Conversely, IL-1β can be methylated and regulated at a single CpG, −299 bp (Hashimoto et al., 2013). DNMTs can also both be regulated by and interact with the cell cycle regulators p53/Sp1, MDM, pRb, and E2F (Robertson et al., 2000; Kimura et al., 2003; Esteve et al., 2005; Lin et al., 2010).

## TETs

The TETs are down-regulated in the placenta, just like in cancer cells, which means 5hmC is reduced in the DNA of cells and the reduction in gene body-associated 5hmC generally inhibits gene expression (Ficz et al., 2011; Lian et al., 2012; Pfeifer et al., 2013). TET1 partly suppresses invasion in cancer by binding to *TIMP2/3* and demethylating the promoters to activate *TIMP2/3*, and in turn down-regulates *MMP* expression (Hsu et al., 2012). Conversely, depletion of TET1 hypermethylates and silences *TIMP2/3* (Hsu et al., 2012). The TETs are the proposed active DNA demethylases that convert 5mC to 5hmC, and then can continue that pathway so that eventually 5mC is converted to cytosine (Seisenberger et al., 2013). There has been a suggestion that oxygen regulation of gene expression is mediated by TET conversion of 5mC to 5hmC (Thalhammer et al., 2011). Recently it has been reported that under redox conditions DNMT3A and DNMT3B, as well as being methylases, can demethylate DNA, such as in cancer (Kangaspeska et al., 2008; Metivier et al., 2008; Chen et al., 2012). Perhaps DNMT3A and DNMT3B can, under hypoxic conditions in the endometrium, aberrantly demethylate DNA in EVTs?

## Conclusion

If any of the epigenetic enzymes of DNMTs, TETs, HDACs, and ncRNAs are dysfunctional in EVTs then the differentiation and invasiveness of EVTs may be compromised (Rugg-Gunn, 2012; Fu et al., 2013; Ji et al., 2013; Mouillet et al., 2013). Environmental factors, such as diet or oxygen stress, can disrupt epigenetic regulation of trophoblasts (Novakovic and Saffery, 2012), for example, hypoxia of <1% oxygen in STBs can affect the AP-1 transcription factor and adversely disrupt the function of DNMT1 (Yuen et al., 2013). Poorly functioning trophoblasts fail to provide an adequate blood supply in circulation through the placenta to the fetus and this has been linked to pathologies of pregnancy such as preeclampsia and IUGR (Maccani and Carmen, 2009; Kokkinos et al., 2010). Future work will attempt to understand the interactive nature of all the epigenetic mechanisms together and their effect on the complex biological system of trophoblast differentiation and EVT invasion in normal as well as pathological conditions.

### Conflict of interest statement

The authors declare that the research was conducted in the absence of any commercial or financial relationships that could be construed as a potential conflict of interest.
